# Efficacy of controlled‐release dinoprostone vaginal insert for elective induction of labor before due date

**DOI:** 10.1111/jog.16123

**Published:** 2024-10-13

**Authors:** Junko Tamai, Satoru Ikenoue, Keisuke Akita, Yuka Fukuma, Yuya Tanaka, Keita Hasegawa, Toshimitsu Otani, Yoshifumi Kasuga, Mamoru Tanaka

**Affiliations:** ^1^ Department of Obstetrics and Gynecology Keio University School of Medicine Shinjuku Tokyo Japan

**Keywords:** before due date, controlled‐release PGE_2_ vaginal insert, dinoprostone, labor induction, vaginal delivery

## Abstract

**Aim:**

The induction of labor before due date has recently been proved to reduce the rate of cesarean sections and is not associated with increased risk of adverse perinatal outcomes as compared to expectant management. Controlled‐release dinoprostone (PGE_2_) vaginal insert has recently been approved for use in Japan. However, evidence regarding its efficacy in cervical ripening and labor induction before due date remains limited. We aimed to compare the efficacy of PGE_2_ vaginal inserts and mechanical dilation for labor induction before due date.

**Methods:**

This retrospective cohort study included 206 mothers at 37, 38, and 39 weeks' gestation delivered at our institution between January 2021 and October 2022. Perinatal outcomes, including the success rate of vaginal delivery, were compared between the PGE_2_ (*n* = 46) and metreurynter/laminaria tent (non‐PGE_2_) (*n* = 160) groups. The success rate of vaginal delivery was defined as the proportion of women who delivered vaginally within 48 h of initiating oxytocin augmentation.

**Results:**

The success rate of vaginal delivery was significantly higher in the PGE_2_ group (37/49, 80.4%) than in the non‐PGE_2_ group (106/177, 66.2%). Emergency cesarean section related to non‐reassuring fetal status was performed with none in the PGE_2_ group and with eight (5.0%) in the non‐PGE_2_ group.

**Conclusions:**

The rate of vaginal delivery was significantly higher in the PGE_2_ group for elective labor induction between 37 and 39 weeks. The PGE_2_ vaginal insert could increase the success rate of vaginal delivery for elective induction of labor at 39 weeks.

## INTRODUCTION

The induction of labor is a common obstetric intervention, conducted in approximately 20%–30% of the pregnant women in recent years.^1^, ^2^, ^3^ It stimulates labor before its spontaneous onset to promptly achieve vaginal delivery. A recent randomized controlled study showed that the induction of labor at 39 weeks of gestation in low‐risk nulliparous women can result in a lower frequency of cesarean delivery and is not associated with an increased risk of adverse perinatal outcomes compared with expectant management.^1^

The success rate of vaginal delivery in labor induction is known to be decreased with an unfavorable cervix, especially for mothers with a Bishop score^4^ of less than 7.^2^, ^5^, ^6^ Hence, pharmacological or mechanical cervical ripening is usually required before labor induction, especially for mothers undergoing it before due date. Prostaglandin E_2_ (PGE_2_) is a cytokine that plays key roles in cervical ripening and parturition. During pregnancy, PGE_2_ is usually produced in the cervix, uterus, placenta, and fetal membranes.^5^, ^7^ The vaginal administration of PGE_2_ may lead to changes in the cervix that mimic the natural cervical ripening process (i.e., dissolution of collagen fibrils and increased water content in the cervix). As a result, the cervix becomes softened and distensible and is, therefore, more amenable to thinning and dilation.^7^ PGE_2_ has also been reported to sensitize the myometrium to the effects of endogenous or exogenous oxytocin, by stimulating endogenous prostaglandin F_2α_ production.^5^, ^8^

Vaginal administration of PGE_2_ is used in the United States, European Union, and several other countries worldwide for cervical ripening. PGE_2_ vaginal insert (PROPESS®) slowly releases PGE_2_ and promotes cervical ripening. It was approved for use in Japan in 2020. However, evidence regarding its efficacy in cervical ripening and labor induction before 40 weeks of gestation remains limited. Meanwhile, since the COVID‐19 pandemic (2020 to 2022), planned vaginal delivery before due date has been promoted for all women at our institution to help prevent nosocomial infections.^9^ Using this standardized change in protocol during that period, we aimed to compare the efficacy of PGE_2_ vaginal inserts and mechanical dilation for labor induction before 40 weeks of gestation.

## METHODS

This retrospective cohort study was conducted involving 206 singleton pregnancies with an unfavorable cervix (Bishop score <7) who were scheduled for labor induction before due date (37–39 weeks of gestation) at Keio University Hospital between January 2021 and October 2022. Mothers with prelabor rupture of membranes, hypertensive disorders in pregnancy, gestational diabetes, or those receiving medication for threatened preterm delivery were excluded from the study. Induction of labor was planned on the discretion of the attending physicians considering maternal parity and cervical opening. Only those women who agreed to use PGE_2_ for cervical ripening was allocated to PGE_2_ group. In addition, each obstetrician chose whether to use PGE_2_ or not. A PGE_2_ vaginal insert was used in 46 women (PGE_2_ group), whereas a metreurynter (inflated with 40 mL) or laminaria tent was used in 160 women (non‐PGE_2_ group).

Fetal heart rate monitoring was initiated before the placement of the PGE inserts in the posterior vaginal fornix to confirm the absence of non‐reassuring fetal status or uterine hyperstimulation. While the PGE_2_ vaginal insert is placed in the vagina, continuous fetal heart rate monitoring and checking of the maternal condition is required to avoid side effects. To reduce the risk of unexpected perinatal complications in the night shift, PGE_2_ was inserted soon after admission and removed after 6 h (at the end of the daytime) in our institution for safety reasons. It was removed immediately if the mother experienced regular uterine contractions with pain within 3‐min intervals or presented with complications, such as rupture of the membrane or other maternal side effects, including nausea and vomiting. If further cervical ripening was required, mechanical cervical ripening using a metreurynter or laminaria tent was performed more than 1‐h interval after the removal of PGE_2_. Labor was induced using oxytocin the following day.

Maternal characteristics and perinatal outcomes, including the success rate of vaginal delivery, were compared between the groups. The success rate of vaginal delivery was defined as the proportion of women who delivered vaginally within 48 h of initiating oxytocin administration. This is due to the institutional clinical practice; if mothers who did not get in labor within 48 h (considered as induction failure), they discharged from the hospital and re‐admission for planned delivery was planned next week. Chorioamnionitis was diagnosed based on the criteria reported by Lencki et al.^10^, ^11^ Between‐group comparisons were performed using the chi‐square test or unpaired *t*‐test. All statistical analyses were performed using SPSS version 27.0 (SPSS Inc., Chicago, IL), with statistical significance determined at *p* < 0.05. The study was approved by the Ethics Committee of Keio University School of Medicine (No. 20150103). As all information was anonymous in the institutional database, obtaining informed consent from each included woman was not needed.

## RESULTS

The maternal characteristics and perinatal outcomes are summarized in Table 1. Maternal age, parity, pre‐pregnancy body mass index, gestational weight gain, gestational age, the Bishop score at cervical ripening, cervical dilation before and after cervical ripening, birth weight, and umbilical artery blood pH were comparable between the PGE_2_ and non‐PGE_2_ groups.

**TABLE 1:** Maternal characteristics and perinatal outcomes (*N* = 206).

Characteristics	PGE_2_ group (*N* = 46)	Non‐PGE_2_ group (*N* = 160)	*p*‐Value
Age, years	34.9 ± 4.8	34.0 ± 4.6	0.13
Pre‐pregnancy BMI, kg/m^2^	20.7 ± 2.4	21.1 ± 3.1	0.45
Primiparous	39 (85%)	118 (74%)	0.44
Method of conception			0.23
Natural insemination	36 (78%)	113 (71%)	
IVF‐ET	10 (22%)	47 (29%)	
Gestational weeks at delivery	39.3 ± 0.75	38.9 ± 0.76	0.07
37 weeks	3 (6.5%)	17 (10.6%)	0.011
38 weeks	13 (28.3%)	76 (47.5%)	
39 weeks	30 (65.2%)	67 (41.9%)	
Bishop score	2.0 ± 1.6	2.1 ± 1.6	0.22
Cervical dilation before cervical ripening (cm)	1.0 ± 0.7	1.4 ± 1.0	0.05
Cervical dilation after cervical ripening (cm)	1.9 ± 1.5	2.0 ± 1.6	0.63
Birth weight (g)	3104 ± 326	3036 ± 304	0.10
Apgar score (1 min)	8.1 ± 0.67	7.9 ± 1.0	0.09
Apgar score (5 min)	8.9 ± 0.6	8.8 ± 0.7	0.08
Umbilical artery blood pH	7.31 ± 0.48	7.30 ± 0.54	0.34

*Note*: Data are presented as mean ± SD or *N* (%).

Abbreviations: BMI, body mass index; IVF‐ET, in vitro fertilization‐embryo transfer.

The success rate of vaginal delivery was significantly higher in the PGE_2_ group (78.2%) than in the non‐PGE_2_ group (59.4%) (*p* < 0.05) (Table 2). This result remained significant after excluding 19 mothers who required both PGE_2_ and mechanical dilation (metreurynter or laminaria tent) (*p* = 0.027). PGE_2_ inserts were removed within 6 h in 18 women (in 16 due to uterine hyperstimulation or in labor and in 2 due to rupture of the membrane). The total rate of vaginal delivery (including vaginal delivery that took more than 48 hours after initiating oxytocin administration or required re‐admission) was also significantly higher in the PGE_2_ group as compared to the non‐PGE_2_ group (*p* = 0.019). The duration of delivery (duration of oxytocin administration) was also significantly shorter in the PGE_2_ group (7.9 ± 11.3 h) as compared to the non‐PGE_2_ group (15.2 ± 13.5 h) (*p* = 0.003). The post‐hoc analyses were performed to compare the success rate of vaginal delivery at each gestational week. The success rate of vaginal delivery was significantly higher in the PGE_2_ group especially at 39 weeks (*p* = 0.009), and not at 37 and 38 weeks. When the mothers were sub‐grouped by the parity, the success rate of vaginal delivery was also significantly higher in the PGE_2_ group in the primiparous mothers (*p* = 0.005). Emergency cesarean section related to non‐reassuring fetal status was performed in none of the women in the PGE_2_ group and eight (5.0%) women in the non‐PGE_2_ group. Those who were performed cesarean section due to maternal chorioamnionitis were none in the PGE_2_ group, while two (1.2%) in the non‐PGE_2_ group (Table 2).

**TABLE 2:** Mode of delivery and indications of emergency cesarean section.

	PGE_2_ group (*n* = 46)	Non‐PGE_2_ group (*n* = 160)	*p*‐Value
Successful vaginal delivery	36 (78.2%)	95 (59.4%)	0.013
at 37 weeks	3/3 (100%)	10/17 (58.8%)	0.484
at 38 weeks	10/13 (76.9%)	48/76 (63.2%)	0.316
at 39 weeks	23/30 (76.7%)	39/67 (58.2%)	0.009*
primiparous	29 (76.3%)	58 (50%)	0.005
Emergency cesarean section	9 (19.6%)	51 (31.9%)	0.039
Indications			0.676
Arrested labor	7 (15.2%)	33 (20.6%)	0.458
Non‐reassuring fetal status	0 (0.0%)	8 (5.0%)	0.294
Malrotation	1 (2.2%)	2 (1.2%)	0.356
Chorioamnionitis	0 (0.0%)	2 (1.2%)	0.748
Hypertensive disorder	1 (2.2%)	4 (2.5%)	0.526
Others	0 (0.0%)	2 (1.2%)	0.748

*Note*: Successful vaginal delivery was defined as vaginal delivery within 48 hours after the initiation of oxytocin administration.

## DISCUSSION

The present study examined the efficacy of PGE_2_ vaginal inserts for labor induction before due date. The main finding revealed that the women in the PGE_2_ group exhibited a significantly higher rate of vaginal delivery without increasing the risk of cesarean section due to non‐reassuring fetal status than that of the women in the non‐PGE_2_ group.

Prior to labor induction, cervical ripening is crucial for successful vaginal delivery. However, the most effective method for cervical ripening remains unclear. A previous study including 397 women demonstrated no statistical difference in the proportions of vaginal delivery within 24 h between the PGE_2_ vaginal insert and Foley catheter groups.^5^ Another study reporting the use of PGE_2_ vaginal insert for cervical ripening at 41 weeks of gestation revealed equal success rates of vaginal delivery between the PGE_2_ and non‐PGE_2_ groups.^12^ Several studies, including substantial ratios of post‐term pregnancies, reported similar rates of cesarean delivery and maternal and neonatal adverse events in the PGE_2_ and mechanical cervical dilation groups.^13^ In contrast, Cromi et al. reported that a greater proportion of women achieved vaginal delivery within 24 h when treated with a double‐balloon catheter, as compared to those treated with the PGE_2_ vaginal insert.^14^ However, in another study, Yamaguchi et al. analyzed findings from women who required labor induction based on medical indication at a mean gestational age of 39.4 weeks and reported a higher rate of vaginal delivery in the PGE_2_ group than in the non‐PGE_2_ group.^15^ Considering the findings of previous studies and the present study, PGE_2_ may be particularly useful for the inducing labor before due date.

In the present study, the PGE_2_ group demonstrated a higher rate of vaginal delivery than that of the non‐PGE_2_ group, whereas cervical dilation and the Bishop score before cervical ripening did not differ between the two groups. PGE_2_ plays key roles in parturition by inducing changes in the cervix that mimic the natural cervical ripening process. Additionally, it enhances the responsiveness of the myometrium to endogenous and exogenous oxytocin.^5^, ^8^ This possibly explains the findings of the present study that PGE_2_ increased the success rate of vaginal delivery using oxytocin augmentation, even though the Bishop scores before cervical ripening were comparable between the two groups.

The proportion of pregnant women requiring emergency cesarean section due to non‐reassuring fetal status was significantly lower in the PGE_2_ group than in the non‐PGE_2_ group. Several previous reports indicated that prostaglandins are associated with uterine tachysystole and hyperstimulation, leading to fetal heart rate abnormalities.^16^, ^17^ In a previous study, the rate of emergency cesarean section because of fetal non‐reassuring status with the ripening agent in situ was reported to be significantly higher in the PGE_2_ vaginal insert group than in the Foley catheter group.^16^ In contrast, Yamaguchi et al. observed no increased risk of uterine hyperstimulation and fetal heart rate abnormalities in the PGE_2_ group.^15^ The present study also revealed no difference in the cesarean section rate or incidence of perinatal complications between the two groups, similar to the findings of Wang et al.^8^ This finding may be attributed to the prompt removal of the PGE_2_ vaginal insert upon detecting uterine hyperstimulation. Indeed, the PGE_2_ vaginal insert was removed in less than 6 h in 22 (47.8%) women in our study, which could have reduced the risk of uterine hyperstimulation, fetal heart rate abnormalities, and emergency cesarean section. Meanwhile, in the present study, to reduce the risk of unexpected perinatal complications during the night shift, PGE_2_ was inserted soon after admission and removed at the end of the daytime in our institution for safety reasons. Nevertheless, PGE_2_ group showed significantly higher rate of vaginal delivery, which indicates placing PGE_2_ for 6 h possibly be effective to stimulate cervical ripening and increase the success rate of vaginal delivery.

Additionally, the incidence of chorioamnionitis was relatively lower in the PGE_2_ group than in the non‐PGE_2_ group (0% vs. 1.2%). The duration of labor has been directly associated with increased risks of chorioamnionitis, postpartum fever, and neonatal infection.^8^, ^17^, ^18^ A meta‐analysis comparing the effects of PGE_2_ vaginal insert and the Foley catheter revealed that the mean time from treatment initiation to delivery was significantly shorter for women who received PGE_2_ vaginal insert.^8^ In the present study, no patient required cesarean section due to chorioamnionitis in the PGE_2_ group, which might have been due to the shorter time lapsed between the induction of labor and vaginal delivery.

The strength of our study is that it included a well‐characterized homogeneous cohort of the Japanese population. We excluded women with epidural analgesia or rupture of the membrane before cervical ripening, which could have a major effect on the progression of labor and delivery. Additionally, the adoption of institutional clinical practice policies to minimize the transmission of COVID‐19 led to the induction of labor before due date for all women. This approach ensured the absence of selection bias, except for the exclusion criteria determined a priori.

The limitations of this study included its retrospective nature and the selection bias. Because PGE_2_ has just approved to use in Japan in this study period, only those women who agreed to use PGE_2_ for cervical ripening was allocated for PGE_2_ group. In addition, each obstetrician chose whether to use PGE_2_ or not, and induction of labor was planned on the discretion of the attending physicians considering maternal parity and cervical ripening. These reasons possibly caused the unbalance of the allocation of each group. However, in spite of these possible selection bias, there was no significant difference in maternal background between the PGE_2_ group and non‐PGE_2_ group as shown in Table 1. Especially, the cervical dilation and Bishop scores before cervical ripening did not differ between the two groups. Furthermore, since the Bishop scores were evaluated by physicians possibly with significant interobserver differences, measurement errors may have affected the present results.

The additional mechanical dilation for cervical ripening could affect the result, however, the success rate of vaginal delivery remained significantly higher in the _2_ group after excluding 19 cases who required instrumental ripening after removing the PGE_2_ vaginal insert.

There are still a few institutions that conduct induction of labor before due date because previous studies have suggested to avoid elective labor induction due to increased risk of cesarean section and perinatal complications.^19^ However, Grobman et al. in a randomized controlled trial have shown that labor induction at 39 weeks of gestation is not associated with a higher risk of adverse perinatal outcomes than that of expectant management.^1^ The present study showed the success rate of vaginal delivery was significantly higher in the PGE_2_ group especially at 39 weeks, which supports this previous study.

In conclusion, vaginal administration of PGE_2_ significantly increased the rate of vaginal delivery in elective labor induction at 39 weeks without increasing the risk of cesarean section due to non‐reassuring fetal status. The PGE_2_ vaginal insert could be effective to low‐risk women for cervical ripening and induction of labor at 39 weeks, which may contribute to improved perinatal outcomes.

## CONFLICT OF INTEREST STATEMENT

The authors report there are no competing interests to declare. Dr. Yoshifumi Kasuga is an Editorial Board member of JOG Journal and a co‐author of this article. To minimize bias, they were excluded from all editorial decision‐making related to the acceptance of this article for publication.

## Data Availability

The data that support the findings of this study are available on request from the corresponding author. The data are not publicly available due to privacy or ethical restrictions. This study was conducted using institutional data with the approval of the institutional ethics committee. The institutional ethics committee has not approved to provide the data to a third party, and separate permission from the ethics committee is required for data to be publicly shared.
